# Study of brain network alternations in non-lesional epilepsy patients by BOLD-fMRI

**DOI:** 10.3389/fnins.2022.1031163

**Published:** 2023-01-18

**Authors:** Zhisen Li, Xiaoxia Hou, Yanli Lu, Huimin Zhao, Meixia Wang, Bo Xu, Qianru Shi, Qian Gui, Guanhui Wu, Mingqiang Shen, Wei Zhu, Qinrong Xu, Xiaofeng Dong, Qingzhang Cheng, Jibin Zhang, Hongxuan Feng

**Affiliations:** ^1^Department of Radiology, The Affiliated Suzhou Hospital of Nanjing Medical University (Suzhou Municipal Hospital), Suzhou, Jiangsu, China; ^2^Department of Neurology, The Affiliated Suzhou Hospital of Nanjing Medical University (Suzhou Municipal Hospital), Suzhou, Jiangsu, China

**Keywords:** epilepsy, BOLD-fMRI, graph theory analysis, independent component analysis, brain network

## Abstract

**Objective:**

To investigate the changes of brain network in epilepsy patients without intracranial lesions under resting conditions.

**Methods:**

Twenty-six non-lesional epileptic patients and 42 normal controls were enrolled for BOLD-fMRI examination. The differences in brain network topological characteristics and functional network connectivity between the epilepsy group and the healthy controls were compared using graph theory analysis and independent component analysis.

**Results:**

The area under the curve for local efficiency was significantly lower in the epilepsy patients compared with healthy controls, while there were no differences in global indicators. Patients with epilepsy had higher functional connectivity in 4 connected components than healthy controls (orbital superior frontal gyrus and medial superior frontal gyrus, medial superior frontal gyrus and angular gyrus, superior parietal gyrus and paracentral lobule, lingual gyrus, and thalamus). In addition, functional connectivity was enhanced in the default mode network, frontoparietal network, dorsal attention network, sensorimotor network, and auditory network in the epilepsy group.

**Conclusion:**

The topological characteristics and functional connectivity of brain networks are changed in in non-lesional epilepsy patients. Abnormal functional connectivity may suggest reduced brain efficiency in epilepsy patients and also may be a compensatory response to brain function early at earlier stages of the disease.

## Introduction

Epilepsy is a chronic seizure disorder characterized by abnormal neuronal activity causing recurrent neurological disturbances (Lv et al., 2014). Currently, the incidence of epilepsy is about 1–2% worldwide (Falco-Walter, 2020). Long-term recurrent seizures can lead to functional impairment, cognitive decline and mental abnormalities, seriously affecting patients’ health and quality of life. The 2017 edition of the International League Against Epilepsy Guidelines divides epilepsy etiologies into 6 categories: structural, genetic, infectious, metabolic, immune, and unknown etiology (Fisher, 2017). The diagnosis of epilepsy relies on clinical, electrophysiological, and imaging techniques. Non-lesional epilepsy (NLE) is defined as a type of epilepsy without definite lesions in conventional imaging examinations such as CT and MRI (Zhang et al., 2011), so its clinical etiological diagnosis is relatively difficult.

As recent medical imaging technology is developing rapidly, resting state functional magnetic resonance imaging (rs-fMRI) has been increasingly used in brain network research (Songjiang et al., 2021). Blood oxygen level dependent (BOLD) imaging is an emerging functional imaging technique that indirectly reflects the temporal and spatial distribution of neuronal activity in the brain by detecting changes in oxygen levels in the brain tissue, thus reflecting changes in the neural network of the brain tissue (Detre, 2006). At present, scholars increasingly recognize that the human brain is composed of a complex functional network with topological properties. Through graph theory analysis (GTA), the complete brain can be divided into different nodes and edges, so as to construct the structure or function network of the brain (Zhang et al., 2010b). Independent component analysis (ICA) can extract different functional brain network components in the resting state on the basis of data-driven model. The above methods are now widely used to analyze brain networks in a variety of central nervous system disorders (Sendi et al., 2020; Liu et al., 2021).

In our research, we studied the topological properties of brain networks in NLE patients based on BOLD-fMRI, and further analyzed the functional connectivity between brain networks. By understanding the interconnections between different brain networks and the abnormal changes that may be caused by these connections, we can explore the underlying pathophysiological mechanisms of the occurrence and development of patients with non-focal epilepsy.

## Materials and methods

### Subjects

Twenty-six NLE patients diagnosed by the Department of Neurology, Suzhou Hospital Affiliated to Nanjing Medical University, from June 2018 to April 2022 were enrolled in this study (1 patient was excluded because of excessive head movement). Inclusion criteria: (1) patients met the 2017 international league against epilepsy (ILAE) epilepsy diagnostic criteria (Fisher et al., 2017); (2) no intracranial lesions were found on routine imaging examination; (3) without other physical or mental diseases; (4) right-handed; (5) no contraindications to MRI examination. All patients were clinically assessed by a detailed epileptic history, neurological and psychiatric evaluation, electroencephalogram and cranial MRI. Forty-two normal healthy volunteers matched to the epilepsy group in terms of sex, handedness, age, and education were recruited as controls. All healthy controls were proved without neuropsychological history and definite intracranial lesions on routine MRI. All subjects were assessed for neuropsychological and cognitive status by the Hamilton Anxiety Scale (HAMA), the Hamilton Depression Scale (HAMD), and the Montreal Cognitive Assessment (MoCA). The study was approved by the Ethics Committee of Suzhou Hospital Affiliated to Nanjing Medical University, and written informed consent was obtained from each subject.

### Image acquisition

Routine MRI sequences and resting-state BOLD sequences were performed using a Siemens Skyra 3.0T MR scanner with an 8-channel head coil. All subjects remained closed-eyed, awake and quiet during the examination. Conventional MRI sequences included transverse T1WI, T2WI, FLAIR, and DWI, sagittal T2WI. BOLD-fMRI parameters: TR:2720 ms, TE:30.0 ms, layer thickness: 3.0 mm, layer spacing: 0 mm; FOV:260 mm × 260 mm, matrix :192 × 192, turning Angle 2°, 15°.

### Data preprocessing

We used the DPABI toolbox version 4.0 to preprocess fMRI images of all subjects. The specific process was as follows :(1) removal of the acquisition data at the first 5 time points; (2) time layer correction; (3) removal of images of subjects with head movements exceeding 3 mm or 3° by head movement correction; (4) Dartel alignment; (5) removal of linear drift in the BOLD signal; (6) regression of white matter signal, cerebrospinal fluid signal and Friston24 head motion parameters; (7) band-pass filtering (0.01–0.08 HZ).

### Brain network construction and topological properties

The brain network nodes were defined using the AAL90 template. The AAL90 template divided the brain into 90 regions, each brain region was considered as a node in the brain network, and functional connections were defined as edges. The time series of each brain region were extracted from the preprocessed functional data, and the Pearson correlation coefficients were calculated for the BOLD time series between brain regions. Fisher’s Z-transformation was done on the correlation coefficients as the connected edge values between the nodes in the network, so that a 90*90 network matrix was obtained for each subject. The graphic model of the brain functional network was constructed by a binary connection matrix. The lower limit of sparsity was automatically estimated according to Gretna software (Watts and Strogatz, 1998; Wang et al., 2015), and the upper limit of sparsity was selected according to the small-world property sigma > 1.1 for more than 90% of the subjects. The topological properties including global efficiency (E_glob_), local efficiency (E_loc_), clustering coefficient (C_*p*_), shortest path length (L_*p*_), normalized clustering coefficient (γ), normalized shortest path length (λ), small-worldness parameters (σ)over network sparsity thresholds of 5%–28% with an increment of 1% were analyzed. We calculated the area under the curve (AUC) values of each index, and finally analyzed the AUC values for comparison between groups.

### Functional network connectivity (FNC)

**FIGURE 1:**
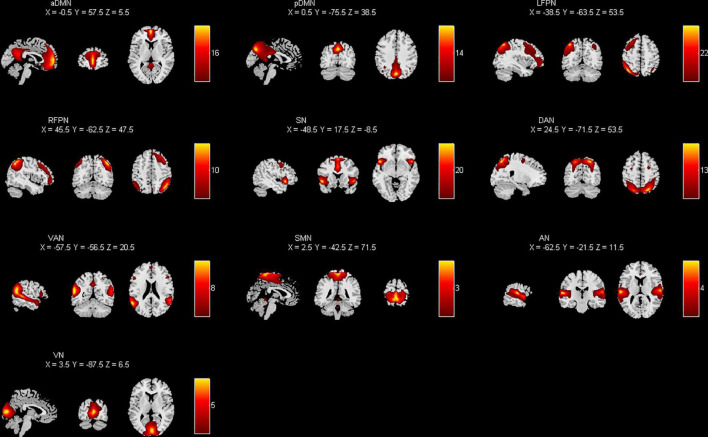
Spatial distribution of each brain network component by applying independent component analysis (ICA) extraction.

The pre-processed data were decomposed into functional networks using the grouped ICA component of the Functional Magnetic Resonance Toolbox (GIFT v4.0a).^1^ The software automatically calculated 31 independent components. Prior to ICA decomposition, the data were subjected to a dimensionality reduction operation using the principal component analysis method. To ensure the stability of the results, we repeated the ICA 20 times using ICASSO tool. Finally, the spatial distribution maps and time series of independent components for each individual subject were obtained using the GICA reverse reconstruction method. The components were selected empirically from the results: (1) high activation regions of functional network components should be in the gray matter and have less overlap with cerebrospinal fluid, white matter, and ventricles (2) time series of functional network components were dominated by low frequency components. As shown in Figure 1, the 10 network components of interest included the anterior default mode network (aDMN), posterior default mode network (pDMN), left frontoparietal network (LFPN), right frontoparietal network (RFPN), salience network (SN), dorsal attention network (DAN), ventral attention network (VAN), sensorimotor network (SMN), auditory network (AN), and visual network (VN).

The time series of each interesting component were obtained for post-processing using the Mancovan toolbox in GIFT. Noise reduction was performed on the components of the above 10 networks by first de-linearizing the time series and then filtering them with a low-pass filter with a high frequency cutoff of 0.15 Hz. Finally, the Pearson correlation coefficients were calculated for the time series after the noise reduction of the independent components to construct the FNC matrix.

### Statistical analysis

Statistical analysis was performed by SPSS22.0. The measurements that conformed to a normal distribution (e.g., age, years of education, HAMA scores, HAMD scores, MoCA scores) were compared by independent sample *t*-tests. The enumeration data expressed as frequencies or percentages were compared using χ^2^-test. The AUC values of brain network topology indicators, the edge-connected functional connectivity matrices, and the FNC matrices were compared using two independent samples *t*-tests. *P* < 0.05 indicated statistically significant difference.

## Results

### Subjects’ baseline information

There were 26 epilepsy patients in total participated in our research, of which 1 was finally excluded due to poor quality of cranial magnetic resonance imaging. All enrolled epileptic patients’ clinical information were listed in Table 1. There were no differences in age, gender, years of education, anxiety and depression assessment and cognitive assessment between two groups (Table 2).

**TABLE 1:** Clinical information of non-lesional epilepsy (NLE) patients.

Index	Age (years)	Gender	Education (years)	Epilepsy duration (years)	Seizure frequency (per year)	Seizure type	Antiepileptic drugs kinds	HAMA score	HAMD score	MoCA score
1	34	F	16	2	0	GTCS	1	28	22	29
2	32	M	18	3	1	GTCS	1	33	18	28
3	29	F	10	5	0	FS	1	28	23	28
4	31	F	14	6	1	FS	1	33	14	30
5	28	F	17	3	2	FS	1	22	17	28
6	28	M	14	5	1	GTCS	1	32	25	27
7	27	M	13	2	0	FS	1	26	16	28
8	35	F	14	1	3	FS	2	33	14	27
9	38	F	19	5	0	FS	1	29	19	25
10	40	M	14	6	1	GTCS	2	34	14	23
11	33	F	12	3	0	FS	1	38	28	27
12	30	M	15	4	2	FS	2	30	15	29
13	34	F	16	2	0	FS	1	26	16	30
14	27	M	12	1	1	FS	2	23	24	29
15	33	F	13	5	0	FS	2	29	19	29
16	19	F	16	8	1	FS	1	33	26	24
17	40	F	14	5	0	GTCS	1	26	14	27
18	19	M	16	8	0	GTCS	1	36	16	29
19	40	F	13	10	1	GTCS	2	23	13	28
20	35	F	19	8	0	FS	1	29	19	27
21	25	F	10	2	1	FS	1	26	28	30
22	40	M	14	1	0	FS	1	24	14	26
23	28	M	11	4	0	GTCS	1	21	11	27
24	28	F	18	2	0	FS	1	38	18	28
25	29	F	16	6	1	FS	1	36	16	29

F, female; M, male; GTCS, generalized tonic-clonic seizure; FS, partial seizures.

**TABLE 2:** Comparison of baseline data.

Characteristics	EP (*n* = 25)	HC (*n* = 42)	T/χ^2^ value	*P*-value
Age (years)	31.48 ± 6.27	30.17 ± 5.50	−0.897	0.373
Gender (male/female)	9/16	13/29	0.181	0.670
Education (years)	14.56 ± 2.55	13.88 ± 2.40	−1.094	0.789
Duration of epilepsy (years)	4.44 ± 2.84	–	–	–
HAMA score	29.44 ± 5.00	28.95 ± 4.86	−0.393	0.696
HAMD score	18.36 ± 4.87	16.76 ± 4.42	−1.377	0.173
MoCA score	27.68 ± 1.77	28.14 ± 1.39	1.188	0.239

EP, epilepsy patients; HC, healthy controls.

### Comparison of topological properties of brain networks

In our study, we found that the brain networks of both the epilepsy group and the healthy control group conformed to small-world properties. Within the threshold range, the AUC value of E_loc_ was significantly lower in the epilepsy group (*P* < 0.05); whereas the AUC values of global indicators such as E_glob_, C_*p*_, L_*p*_, γ, λ, and σ were not statistically different between the two groups (*P* > 0.05) (Table 3).

**TABLE 3:** Comparison of the area under the curve (AUC) values of the global indexes.

Index	EP (*n* = 25)	HC (*n* = 42)	T/*x*^2^ value	*P*-value
E_glob_	0.094 ± 0.009	0.096 ± 0.008	1.323	0.190
E_loc_	0.148 ± 0.010	0.153 ± 0.010	2.083	0.041
C_p_	0.119 ± 0.009	0.122 ± 0.007	1.723	0.090
L_p_	0.647 ± 0.077	0.627 ± 0.078	−1.004	0.319
γ	0.500 ± 0.110	0.547 ± 0.128	1.593	0.129
λ	0.270 ± 0.015	0.277 ± 0.014	1.816	0.074
σ	0.419 ± 0.089	0.447 ± 0.110	1.109	0.271

EP, epilepsy patients; HC, healthy controls.

### Edge analysis

**FIGURE 2:**
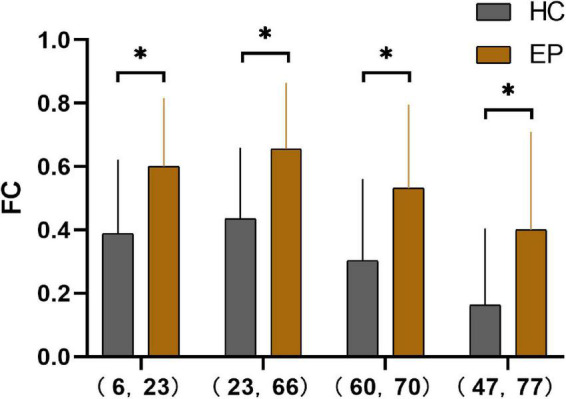
Pearson’s correlation coefficients for four pairs of differential brain regions in the epilepsy group and the healthy control group after Fisher’s Z-transformation *(*P* < 0.001). 6, right orbital superior frontal gyrus; 23, left medial superior frontal gyrus; 66, right angular gyrus; 60, right superior parietal gyrus; 70, right paracentral lobule; 47, left lingual gyrus; 77, left thalamus; HC, healthy controls; EP, epilepsy patients; FC, functional connectivity.

**FIGURE 3:**
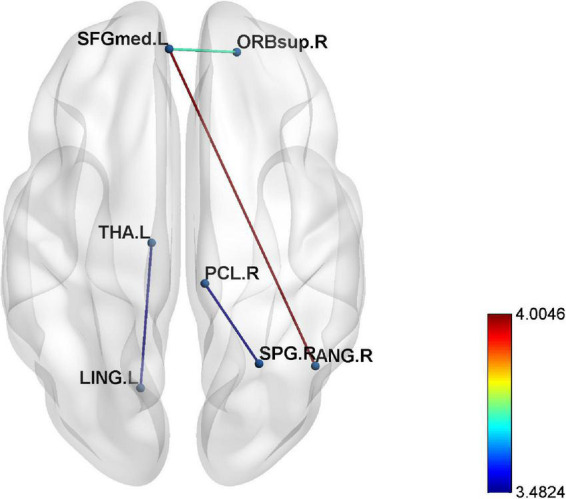
The enhanced four edges connecting seven different regions in epilepsy patients compared to healthy controls (*p* < 0.001).

The epileptic group showed enhanced functional connectivity between the right orbital superior frontal gyrus and left medial superior frontal gyrus, left medial superior frontal gyrus and right angular gyrus, right superior parietal gyrus and paracentral lobule, and left lingual gyrus and thalamus in comparison with healthy controls (*P* < 0.05) (Table 4 and Figures 2, 3).

**TABLE 4:** Group differences in whole-brain functional connectivity.

Encephalic region		T/*x*^2^ value	*P*-value
ORBsup.R (6)	SFGmed.L (23)	3.706	<0.001
SFGmed.L (23)	ANG.R (66)	4.005	<0.001
SPG.R (60)	PCL.R (70)	3.482	<0.001
LING.L (47)	THA.L (77)	3.493	<0.001

ORBsup.R (6), right orbital superior frontal gyrus; SFGmed.L (23), left medial superior frontal gyrus; ANG.R (66), right angular gyrus; SPG.R(60), right superior parietal gyrus; PCL.R(70), right paracentral lobule; LING.L(47), left lingual gyrus; THA.L (77), left thalamus.

### Functional network connectivity (FNC)

**FIGURE 4:**
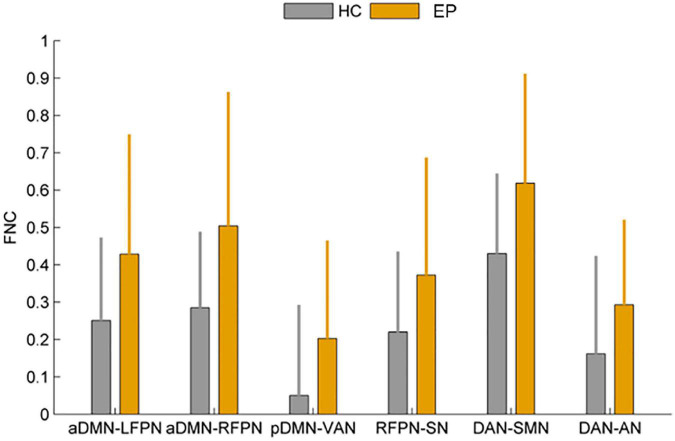
Comparison of functional network connectivity between the epilepsy group and healthy controls (*P* < 0.05). HC, healthy controls; EP, epilepsy patients.

**FIGURE 5:**
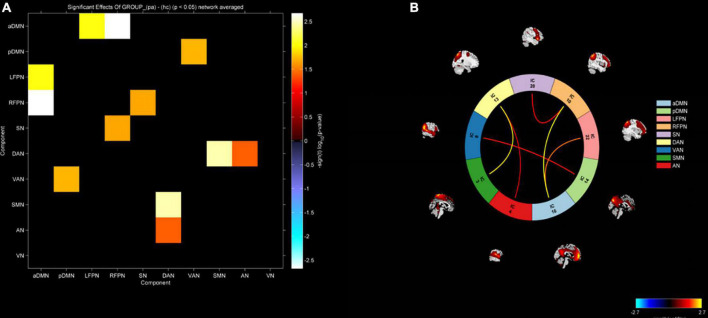
**(A)** Functional network connectivity matrix diagram. **(B)** Functional network connectivity circular graph. Increased functional connectivity between aDMN-LFPN, aDMN-RFPN, pDMN-VAN, RFPN-SN, DAN-SMN, and DAN-AN in the epilepsy group.

In this study, FNC between the anterior default network and left frontoparietal network (aDMN-LFPN), anterior default network and right frontoparietal network (aDMN-RFPN), posterior default network and ventral attention network (pDMN-VAN), right frontoparietal network and projection network (RFPN-SN), dorsal attention network and sensorimotor network (DAN-SMN), and dorsal attention network and auditory network (DAN-AN) were significantly enhanced in NLE patients (*P* < 0.05) (Figures 4, 5).

## Discussion

Due to the lack of clear lesions in routine imaging examinations, the origin of epileptic discharges in the brain of NLE patients cannot be accurately determined, causing certain difficulties for clinical diagnosis and treatment. This study investigated alternations in neural networks by detecting changes in blood oxygen levels in brain tissue in response to neuronal activity based on the principle of BOLD imaging. Through GTA and ICA, two common methods for studying brain networks (Xiao et al., 2019; Sendi et al., 2020; Liu et al., 2021), we explored alternations in topological properties and functional connectivity of brain networks in epilepsy patients.

Our study showed that the brain network of NLE patients still conforms to the “small world” properties, which is an economical and efficient mode of brain network operation, reflecting the brain’s ability to separate and integrate information. The larger the σ value of the indicator, the better the ability to separate and integrate information. In our study, the AUC values of the “small world” indices such as γ, λ, and σ were not statistically different between the NLE patients and HC group. Reasons for this result may be related to the sample size and the age of enrolled patients. Our study found that the E_loc_ in NLE patients was significantly lower than that of HC group, which was in line with the results of most previous studies (Song et al., 2015; Ji et al., 2017; Lee et al., 2020). E_loc_ reflects the ability of local brain regions to exchange information. Due to the long-term abnormal discharging activity of neurons in NLE patients, neurons in the corresponding area are prone to hypoxia, edema, and necrosis, which eventually lead to brain tissues damage and a corresponding reduction in functional connectivity with adjacent brain regions, resulting in a significant decrease in E_loc_. The E_glob_, C_p_ and L_p_ had no statistical difference between two groups. E_glob_ reflects the information exchange and integration capabilities of the whole brain, which represents the work efficiency of the entire brain tissue. C_p_ reflects how closely one node is connected to other nodes in the brain. L_p_ identifies the optimal and shortest information transfer distance from one node to another. All the global indicators represents the efficiency and ability of information transmission in brain tissue. Bernhardt et al. (2013) prompted that the C_p_ and L_p_ were remarkably increased compared with healthy controls in adult temporal lobe epilepsy. In a comparative study of 25 cases of familial hereditary epilepsy and non-familial hereditary epilepsy, Bharath et al. (2016) found that the C_p_ of familial hereditary epilepsy patients decreased, the L_p_ increased, and the σ decreased. The reasons for the differences in topological properties varying in epilepsy patients are not yet clear. The sample size, age, type of epilepsy, and duration of disease of the enrolled patients may all contribute to the different study results. A study on a rat epilepsy model found that the ability to separate and integrate brain networks is related to the frequency of epileptic seizures, providing indirect evidence that differences in seizure frequency also contributed to the differences in findings (Christiaen et al., 2019). The NLE patients included in our study were relatively young (mean age 31.48 ± 6.27 years), had a short duration of disease (mean duration 4.44 ± 2.84 years) and had better seizure control (seizure frequency less than 1 seizure/year). The brain tissue damage in epileptic patients may be relatively mild, so the impact on the whole brain level was relatively small, and the changes of topological features may not be significant.

Previous researches suggested that epilepsy is a broad brain network disease, and long-term and repeated seizures can lead to alternations in the topological properties of the whole or local brain regions (Park et al., 2019; Tavakol et al., 2019). The neurological activities of the brain are often coordinated completion by multiple brain regions. Damaging to one certain brain region may lead to abnormal functions of other brain regions. Connected edge analysis obtains functional connectivity between brain regions by analyzing the correlation of time series of different brain regions (Zheng et al., 2012). In this study, we found that the functional connections between the right orbital superior frontal gyrus and left medial superior frontal gyrus, left medial superior frontal gyrus and right angular gyrus, right superior parietal gyrus and paracentral lobule, and left lingual gyrus, and thalamus were statistically enhanced than those in HC group. The orbital superior frontal gyrus, medial superior frontal gyrus, and angular gyrus are all important components of DMN. DMN belongs to the resting network, which is activated in the resting state and inhibited when the human brain is active (Buckner and DiNicola, 2019). Wei et al. (2015) found that the right frontal pole also had enhanced connectivity in the DMN of patients with idiopathic generalized epilepsy. Some scholars showed that the change of DMN may be the factor leading to the changes of memory, cognition and other functions in epilepsy patients (Lopes et al., 2014; McCormick et al., 2014). According to a study on atonic epilepsy, DMN dysfunction was the abnormal integration of anatomical structures in the DMN (Luo et al., 2011). As we know, when one brain region is damaged, other brain regions with similar functions may show compensatory activation or enhanced connectivity. The presence of enhanced connectivity of brain regions associated with the DMN in our study also suggested that the DMN is damaged in epileptic patients and that the alteration of core nodes is one of the pathological mechanisms of epileptogenesis (Wang et al., 2017). In this study, the connection between the lingual gyrus and thalamus was also enhanced in NLE patients. A meta-analysis of thalamic function (Barron et al., 2012) showed that thalamic nuclei are involved in the whole process of epilepsy onset and development, which indirectly proves that the thalamus may be an important node in the discharging and transmission of epilepsy.

In addition, we not only explored the functional alterations of a specific network node or brain region in NLE patients, but also investigated the FNC level by ICA. In our study, the FNC between aDMN and LFPN, aDMN and RFPN, pDMN and VAN were significantly enhanced in NLE patients. As mentioned above, DMN is one of the most prominent brain network in the resting state, and its alteration leads to disruption of information exchange and integration functions in the resting brain. LFPN and RFPN are mainly related to attention, memory, language, and other functions. Therefore, the abnormal functional connection between DMN and FPN may be one of the reasons for the decline of memory and language functions in epilepsy patients. Wei et al. (2015) found that the functional connectivity between aDMN and LFPN, aDMN and RFPN, pDMN and RFPN were all enhanced in idiopathic generalized epilepsy patients. In addition, the findings in Bonilha’s research were basically consistent with our above results (Bonilha et al., 2013). However, Zhang et al. (2010a) discovered that the functional connectivity between DMN and other brain regions was significantly reduced in the medial temporal lobe epilepsy patients with hippocampal sclerosis. The authors believed that one of the reasons for the differences may be related to the epileptic seizure type. All epileptic patients in our study had no lesions and a short course of epilepsy, while the patients in Zhang et al.’s (2010a) study all had definite manifestations of hippocampal sclerosis and a long course of epilepsy. A study about generalized idiopathic epilepsy also showed that DMN connectivity strength was associated with onset age and seizure duration (Vaessen et al., 2012). Moreover, we also found that the FNC between DAN and SMN, DAN and AN in the NLE patients were also highly enhanced. As an advanced functional network of the human brain, DAN is mainly involved in the control and regulation of attention. Previous studies have shown that DAN is a goal-task-oriented network of brain (Jimenez et al., 2016). SMN and AN are the “sensors” of the human brain, transmitting information and supervising our response to external stimuli, which functionally forming a good cluster with DAN. Liang et al. (2021) found mesial temporal lobe epilepsy patients within 5 years of onset also had enhanced functional network connectivity between DAN and SMN, and between DAN and AN. This scholar showed that abnormal enhanced functional connectivity may suggest reduced efficiency between brain networks in patients with epilepsy. Therefore, combined with the fact that our enrolled epileptic patients have not yet shown significant neuropsychiatric and cognitive abnormalities, we believe that the enhanced functional connectivity between different brain networks not only reflects their close functional connection, but also is a compensatory response to the patient’s existing brain function.

Our study still had shortcomings. First of all, the sample size was insufficient and the age of the patients enrolled was young, so there may be some bias in the study results. In addition, the types of epilepsy were not classified in this study, so we still need to subdivide the types of epilepsy in the future work.

In conclusion, the topological characteristics and functional connectivity of brain networks are changed in epileptic patients. The abnormal functional connectivity may suggest reduced efficiency between brain networks in epileptic patients and also may be a compensatory response to brain function early in the disease.

## Data availability statement

The original contributions presented in this study are included in the article/supplementary material, further inquiries can be directed to the corresponding author.

## Ethics statement

The studies involving human participants were reviewed and approved by the Ethics Committee of Suzhou Hospital Affiliated to Nanjing Medical University. The patients/participants provided their written informed consent to participate in this study.

## Author contributions

ZL and XH were responsible for data collection and analysis, consulting literature, and article writing. YL and JZ were responsible for post-processing of imaging data. HZ, MW, BX, QS, QG, GW, MS, WZ, QX, XD, and QC were responsible for sorting out clinical data and data collection. HF was responsible for project design, data analysis, and article writing. All authors contributed to the article and approved the submitted version.

## Footnotes

1
http://icatb.sourceforge.net
